# SIRT1 in the brain—connections with aging-associated disorders and lifespan

**DOI:** 10.3389/fncel.2015.00064

**Published:** 2015-03-09

**Authors:** Fanny Ng, Laura Wijaya, Bor Luen Tang

**Affiliations:** ^1^Department of Biochemistry, Yong Loo Lin School of Medicine, National University Health SystemSingapore, Singapore; ^2^NUS Graduate School for Integrative Sciences and Engineering, National University of SingaporeSingapore, Singapore

**Keywords:** aging, cognition, neurodegeneration, SIRT1, metabolism

## Abstract

The silent mating type information regulation 2 proteins (sirtuins) 1 of class III histone deacetylases (HDACs) have been associated with health span and longevity. SIRT1, the best studied member of the mammalian sirtuins, has a myriad of roles in multiple tissues and organs. However, a significant part of SIRT1’s role that impinges on aging and lifespan may lie in its activities in the central nervous system (CNS) neurons. Systemically, SIRT1 influences energy metabolism and circadian rhythm through its activity in the hypothalamic nuclei. From a cell biological perspective, SIRT1 is a crucial component of multiple interconnected regulatory networks that modulate dendritic and axonal growth, as well as survival against stress. This neuronal cell autonomous activity of SIRT1 is also important for neuronal plasticity, cognitive functions, as well as protection against aging-associated neuronal degeneration and cognitive decline. We discuss recent findings that have shed light on the various activities of SIRT1 in the brain, which collectively impinge on aging-associated disorders and lifespan.

## Introduction

The sirtuin family comprises of class III protein deacetylases, which unlike the class I and II histone deacetylases (HDACs), have an obligatory dependency on nicotinamide adenine dinucleotide (NAD) as a cofactor (Blander and Guarente, 2004). Sirtuins deacetylate a wide range of histone and non-histone targets, and influence multiple aspects of cellular and organismal physiology and pathology (Haigis and Sinclair, 2010; Sebastián et al., 2012; Chang and Guarente, 2014; Herskovits and Guarente, 2014). The most prominent of the latter is health and lifespan, and the notion of sirtuin activities being systemically beneficial to multiple organs, particularly in the later years of life, has attracted tremendous interest in recent years (Tang, 2011). The family’s founding member, *Sir2* of *S. cerevisiae*, mediates transcriptional silencing of the yeast mating loci, telomeres and the ribosomal DNA (Imai et al., 2000). Its suppression of the formation of rDNA circles (Sinclair and Guarente, 1997) could play a role in extending yeast’s replicative, but not chronological lifespan (Kaeberlein et al., 1999; Fabrizio et al., 2005). Importantly, yeast *Sir2*’s lifespan prolonging activity is also proposed to be biochemically link to, and with its activation mimicking, the effect of caloric restriction/dietary restriction (CR/DR) (Lin et al., 2000; Anderson et al., 2003; Cohen et al., 2004). CR/DR, a regime of moderate reduction of food intake without causing malnutrition, was known to curb metabolic disease as well as extend lifespan in many animal models (Chung et al., 2013). The notion that CR/DR acts through sirtuin has been intensely investigated and debated (Kaeberlein and Powers, 2007; Cantó and Auwerx, 2009a,b; Guarente, 2013; Park et al., 2013). This putative mechanistic link is one reason for the heightened interest in sirtuins as potential drug targets for pharmacological intervention of metabolic disorders and aging (Baur et al., 2012; Chakraborty and Doss, 2013).

Over-expression or activation of *Sir2* orthologues was also shown to increase lifespan of invertebrate models such as *C. elegans* and *Drosophila* (Tissenbaum and Guarente, 2001; Rogina and Helfand, 2004; Wood et al., 2004). These results have been challenged by more recent works that suggest that the lifespan extension effect was smaller than previously reported (Burnett et al., 2011; Lombard et al., 2011; Viswanathan and Guarente, 2011). The mammalian genome has seven sirtuin paralogues (Michan and Sinclair, 2007) that are differentially localized to the nucleus, cytoplasm and mitochondria. Whether sirtuins are *bona fide* longevity factors in mammals and humans has been unclear (Naiman and Cohen, 2012; Park et al., 2013). *Sirt1*, the mammalian paralogue sharing the highest homology to yeast *Sir2* and the best studied, could be either pro- or anti-survival (for example, cancer promoting) under different contexts. Unlike the case in yeast, earlier experiments with transgenic over-expression or pharmacological activation of SIRT1 in mice improved metabolic parameters later in life (Baur et al., 2006; Alcendor et al., 2007; Milne et al., 2007; Herranz et al., 2010), but did not significantly increase lifespan in mice (Herranz et al., 2010). However, several recent reports have collectively provided strong evidence that sirtuins could indeed prolong the lifespan of mice. Transgenic over-expression of SIRT6 was shown to increase the lifespan of male (but not female) mice, likely through its influence on Insulin-like growth factor 1 (IGF-1) signaling (Kanfi et al., 2012). Recently, SIRT2 was found to increase lifespan of mice hypomorphic for the mitotic checkpoint kinase BubR1, the level of which declines in aging and aging-associated diseases (North et al., 2014). Importantly, mice with brain-specific transgenic over-expression of SIRT1 (BRASTO) have also recently been shown to have an extended lifespan (Satoh et al., 2013), and the specific SIRT1 activator SRT1720 extends lifespan of mice even when these were fed a standard diet (Mitchell et al., 2014).

The aging-delayed and lifespan extension phenotype of brain-specific *Sirt1-*overexpressing mice (Satoh et al., 2013) is particularly interesting, and attested to the notion that SIRT1 may effectively influence aging through its activities in the central nervous system (CNS) neurons. SIRT1’s deacetylation of transcription factors such as the forkhead box class O (FoxO) family members (Brunet et al., 2004; Huang and Tindall, 2007), TP53 (Hasegawa and Yoshikawa, 2008) and nuclear factor қB (NF-қB) (Yeung et al., 2004; Chen et al., 2005) modulates key aspects of stress response and survival pathways in postmitotic neurons. In *C. elegans*, the FoxO orthologue *Daf-16* (Ogg et al., 1997) mediates lifespan extension downstream of the IGF-1 receptor/*Daf-2* (Kimura et al., 1997) mutant in worms, and restoring *Daf-2* expression in worm neurons alone negated the lifespan extension effect of *Daf-2* mutants (Wolkow et al., 2000). This suggest that IGF-1 receptor/*Daf-2* signaling in neurons could be critical for lifespan and that this could be influenced by sirtuins through FoxOs. As the enzymatic activity of SIRT1 is regulated by NAD^+^, it is therefore a key nutrient and redox sensor in the energy expensive brain. SIRT1’s role in regulating the activities of key transcription factors (e.g., peroxisome proliferator-activated receptor gamma (PPAR-γ) and its transcriptional co-activator PPARγ coactivator-1α (PGC-1α) and transducer of regulated CREB2 activity 2 (TORC2)) and enzymes (e.g., phosphoglycerate mutase-1) in energy metabolism in peripheral tissues are well known (Cantó and Auwerx, 2009b; Sugden et al., 2010; Li, 2013; Chang and Guarente, 2014). However, metabolic control in mammals is centrally exerted through sensing of energy status in the hypothalamic neurons in the brain, and SIRT1 activities in these neurons influence the development of aging-associated metabolic disorders (Chang and Guarente, 2014; Toorie and Nillni, 2014). SIRT1’s neuroprotective functions are also well known (Tang, 2009; Zhang et al., 2011b). However, by regulating neurite growth and synaptic processes, SIRT1 was recently shown to also play a role in normal cognitive function and synaptic plasticity (Gao et al., 2010; Michán et al., 2010), which is beyond its better known role in countering cognitive decline and neurodegenerative disease in aging.

In the ensuing paragraphs, we highlight recent findings that elucidated the functions of SIRT1 in the brain, and discuss how disruptions of these functions may underlie aging-associated disorders and lifespan.

## SIRT1 in CNS neurons—role in neurogenesis, neurite growth neuronal network connections

SIRT1 is ubiquitously expressed, but more targeted investigations have revealed high levels of expression in the developing mouse CNS (Sakamoto et al., 2004; Ogawa et al., 2011), adult mouse and human brain (Ramadori et al., 2008; Zakhary et al., 2010) as well as the porcine brain (Shan et al., 2009). Within the adult brain, SIRT1 expression is found in most brain regions, is prominent in neurons of the hippocampus and hypothalamus (Ramadori et al., 2008; Michán et al., 2010; Zakhary et al., 2010), and is mostly nuclear. Brain SIRT1 levels are decreased in aged neurons (Quintas et al., 2012), and are downregulated by a high fat diet (Wu et al., 2006; Heyward et al., 2012) and various neuropathological conditions (Pallàs et al., 2008; Julien et al., 2009). On the other hand, SIRT1 levels in brain neurons could be increased by CR/DR (Satoh et al., 2010; Quintas et al., 2012) and physical exercise (Falone et al., 2012; Revilla et al., 2014). SIRT1’s high levels in the mammalian CNS and the way these change with physiological and pathological stimuli are indicative of functional importance.

SIRT1 activity is known to affect fate determination of neural progenitor cells (NPCs) during development. SIRT1 activity is dependent on the redox state, and under conditions of oxidative stress, SIRT1 promotes differentiation of NPCs towards the astroglia lineage partly by limiting the levels and activity of the pro-neuronal transcription factor Mash-1 (Prozorovski et al., 2008). Differentiating neural cells from 8-oxoguanine DNA glycosylase knock-out mice spontaneously accumulate mtDNA damage and concomitantly shift their differentiation direction toward an astrocytic lineage, a result of an increased NAD/NADH ratio and SIRT1 activation (Wang et al., 2011). SIRT1 inhibition or silencing was shown by several authors to promote neuronal differentiation (Prozorovski et al., 2008; Zhang et al., 2011a; Liu et al., 2014) and increased neuronal production in both the subventricular zone and the hippocampus (Saharan et al., 2013). Sensory neuron differentiation from progenitors is dependent on SIRT1’s modulation of the acetylation status of Pax3, which acts upstream of Hes1 and Neurog2. SIRT1 silencing increased the level of acetylated Pax3, with consequential decrease in Hes1 (important for stem cell maintenance) and increase in Neurog2 (important for neuronal properties) activities, thus promoting neurogenesis (Ichi et al., 2011). In a different context, however, pro-neurogenic Bcl6 was shown to promote neurogenesis through SIRT1 recruitment, with the silencing of Hes-5 downstream of Notch signaling (Tiberi et al., 2012). Another report has indicated that SIRT1 inactivation in the adult brain resulted in the expansion of oligodendrocyte progenitors that could generate myelinating oligodendrocytes (Rafalski et al., 2013).

Other than neural cell fate, manipulations of SIRT1 levels and activity have also been shown to modulate neuritogenesis—the outgrowth of axons and dendrites. Cytoplasmic SIRT1 enhanced nerve growth factor-induced neuritogenesis in PC12 cells (Sugino et al., 2010). Insulin-induced neurite outgrowth of SH-SY5Y cells is dependent on SIRT1 (Liu et al., 2013b). Primary neurons from transgenic mice overexpressing SIRT1 have enhanced neurite outgrowth and survival apparently via negative regulation of mammalian/mechanistic target of rapamyscin (mTOR) signaling (Guo et al., 2011). SIRT1 has been detected at the axonal growth cone and its activation has a positive influence on both formation and elongation of axons, likely through deacetylation of AKT (Li et al., 2013). Regeneration of peripheral axon after injury is SIRT1-dependent, and SIRT1 targets miR-138, a suppressor of the axonal regeneration process which could in turn reciprocally suppress SIRT1 expression (Liu et al., 2013a). Overexpression of SIRT1 in hippocampal neurons enhanced dendritic arbor complexity, an effect that is mimicked by its activator resveratrol (Codocedo et al., 2012). Brains of *Sirt1* knockout mice was also shown to exhibit a decrease in dendritic branching, branch length, and complexity of neuronal dendritic arbors (Michán et al., 2010).

**Figure 1:**
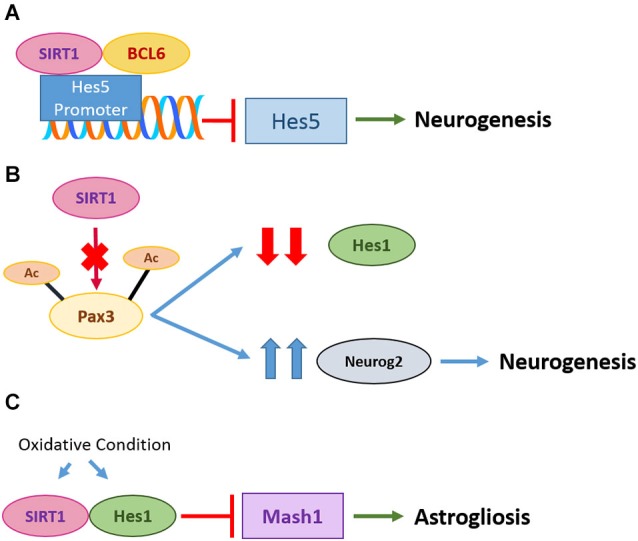
**Roles of SIRT1 in neurogenesis and gliogenesis. (A)** In neural progenitor cells (NPCs), repression of Notch-dependent Hes5 genes by Bcl6 is important for neurogenesis. Bcl6 triggers exclusion of co-activator Mastermind-like 1 and recruits SIRT1 to inhibit transcription of Hes5, promoting neurogenesis. **(B)** Pax3 acetylation on C-terminal lysine residues K437 and K475 is critical for regulation of Hes1 and Neurog2. SIRT1 silencing increased acetylation level of Pax3 and subsequently decreased the promoter activity of Hes1 but increased activity of Neurog2, inducing sensory neuron differentiation. **(C)** Under oxidative condition in NPCs, SIRT1 is upregulated and binds to transcription factor Hes1. This subsequently inhibits pro-neuronal Mash1 and leads to astrogliosis.

Taken together, these results suggest that SIRT1 could influence neuritogenesis of both axon and dendrite via several targets and mechanisms (Figure 1). This would also mean that SIRT1 is likely to have a role, like those documented for the Class I and II HDACs (Gräff and Tsai, 2013), in the connection and plasticity of the neuronal network.

## SIRT1’s role in synaptic plasticity and cognition

SIRT1 has a widely recognized role in neuroprotection and is antagonistic against progression of neurodegenerative processes (Tang, 2009; Srivastava and Haigis, 2011). Its role in synaptic plasticity and cognitive function was therefore more often associated with a reversal of neuropathological conditions that impairs learning and memory. Whether SIRT1 has a role in the physiologically unperturbed versions of learning and memory was clarified only recently. *Sirt1* knockout mice have grossly normal brain anatomy, but exhibited a dendritic development phenotype as noted above (Michán et al., 2010). These mice exhibited defects in synaptic plasticity, although parameters such as basal synaptic transmission are indistinguishable from wild type. Behavioral tests indicated that short-term memory, long term associative memory, as well as spatial learning are all impaired in *Sirt1* knock-outs compared to control. The knock-out mice’s hippocampal Schaffer collateral pathway also exhibited a defect in long term potentiation (LTP).

**Figure 2:**
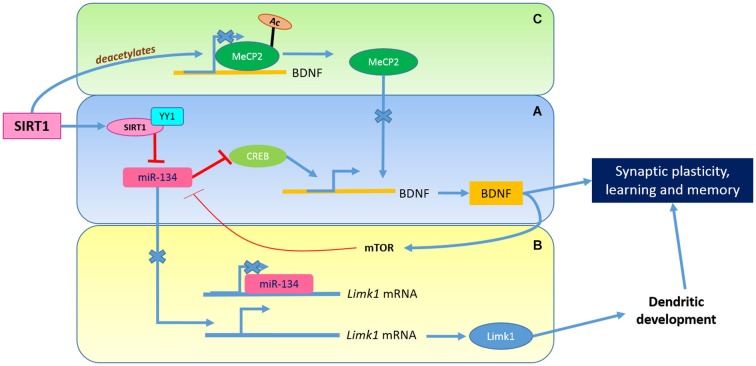
**The Role of SIRT1 in synaptic plasticity**. SIRT1 modulates synaptic plasticity through the regulation of BDNF. The SIRT1 and YY1 complex limits the expression of miR-134 which affects the CREB-BDNF axis **(A)** and leading to enhanced Limk1 protein synthesis **(B)**. The release of BDNF activates mTOR signaling pathway which suppress miR-134 activity. MeCP2 deacetylation by SIRT1 could also promote BDNF transcription **(C)**.

A conditional knockout of SIRT1 in brain (loxP-flanked exon 4 of *Sirt1* with Cre recombinase driven by the *Nestin* promoter) also resulted in a decrease in fear-conditioning and other short-term memory, as well as hippocampal CA1 LTP (Gao et al., 2010). SIRT1-deficient brain had grossly normal anatomy and CA1 neurons had normal basal synaptic transmission. However, the SIRT1-deficient hippocampus had decreased levels of synaptophysin, a marker of the presynaptic termini, and CA1 neurons in these have decreased dendritic spine density. Analysis of known genetic and epigenetic modulators of learning and memory in SIRT1-deficient brain revealed that the levels of a key neurotrophin regulating neural development and synaptic function, the brain-derived neurotrophic factor (BDNF), is reduced. This is explained by a reduction or abolishment of cAMP response element-binding protein (CREB) binding to several BDNF promoters, as the levels of CREB protein (but not mRNA) is downregulated (Figure 2A). The reason why CREB levels are reduced in SIRT1-deficient brain was traced to an upregulation of the levels of miR-134 (which SIRT1 suppresses reciprocally). miR-134 is brain-enriched, and one of its known role is the regulation of dendritic spine size and morphology through modulating the expression of Lim-domain-containing protein kinase 1 (Limk1) (Figure 2B). SIRT1 limits the expression of miR-134 as part of a YYI-containing repressor complex, and promotes BDNF transcription. However, BDNF production may affect mTOR signaling pathway activation, which will also suppress miR-134 activity. The latter also has an effect of relieving miR-134’s repression of Limk1 translation, which promotes dendritic development (Schratt et al., 2006).

SIRT1 could also modulate BDNF expression via the deacetylation of methyl-CpG binding protein 2 (MeCP2; Zocchi and Sassone-Corsi, 2012), a transcription factor mutated in the neurodevelopmental disorder Rett Syndrome (Guy et al., 2011; Liyanage and Rastegar, 2014). BDNF transcription could be elevated by this MeCP2 deacetylation, which promoted its release from the BDNF promoter (Guy et al., 2011; Figure 2C). The presence of high levels of SIRT1, particularly in the hippocampal neurons, may therefore serve a role (amongst others) in synaptic plasticity through the modulation of changes in dendritic spine functions and connections.

From the above discussion, it is easily conceivable that reduction of SIRT1 levels in the aged hippocampus could result in short term memory deficits and cognitive impairment. Aging human adults often exhibit more delocalized brain activity connected to higher order cognitive functions, and recruitment of additional brain areas was proposed as a compensatory mechanism for age-dependent functional decline in the primary areas (Cabeza et al., 2002; Bishop et al., 2010). Aging-associated decline in SIRT1 levels may also impair the plasticity of more distant connections. SIRT1 also has other functions in brain neurons that would contribute to aging and its associated disorders, as further discussed below.

## SIRT1 in hypothalamic neurons—control of energy metabolism, circadian rhythm (and lifespan)

The hypothalamus regulates important aspects of metabolism through its neuroendocrine control of the pituitary gland hormones, and regulates body temperature, hunger/satiety and circadian rhythms, amongst others. Major neurons regulating hunger and food intake in the arcuate nucleus of the hypothalamus are the anorexigenic pro-opiomelanocortin (POMC)-expressing neurons and the orexigenic agouti-related peptide (AgRP) expressing neurons (Morton et al., 2006), and recent findings indicate that SIRT1 has major roles in metabolic homeostasis via its activity in these neurons (Table 1). Inhibition or silencing of hypothalamic SIRT1 in rat (by intracerebro-ventricular administration of inhibitor and siRNA) decreased food intake and body wright via a FoxO1-mediated increase in POMC and decrease in AgRP (Cakir et al., 2009). In rats with diet-induce obesity, brain SIRT1 inhibition increased POMC that leads to an increase in α-melanocyte-stimulating hormone (α-MSH), which could in turn increase thyroid releasing hormone and T3 levels (Cyr et al., 2014).

**Table 1:** **Summary of SIRT1’s roles in metabolic homeostasis**.

No	Model	Manipulation of SIRT1	Observation	Reference
1	POMC expressing neurons	Knockout	Increased α-MSH, increased thyroid hormone and T3 levels, reduced energy expenditure; hence more sensitive to diet-induced obesity	Ramadori et al. (2010, 2011), Cyr et al. (2014)
2	AgRP expressing neurons	Knockout	Decreased response to hunger-inducing hormone ghrelin, reduced food intake	Dietrich et al. (2010)
3	Peripheral tissue neurons	Knockout	Increased insulin sensitivity and insulin receptor	Lu et al. (2013)
4	BSKO mice	Knockout	Defective somatrophic signaling (growth hormone and IGF-1) Defective caloric restriction response	Cohen et al. (2009); Monteserin-Garcia et al. (2013)
5	AgRP expressing neurons	Over-expression	Reduced food intake	Sasaki et al. (2014)
6	POMC expressing neurons	Over-expression	Stimulated energy expenditure	Sasaki et al. (2014)
7	SF-1 expressing neurons	Over-expression	Increased resistance to diet-induced obesity and reduced susceptibility to insulin resistance	Ramadori et al. (2011)
8	Mediobasal hypothalamus	Activated (by resveratrol)	Improved insulin sensitivity	Knight et al. (2011)
9	Mouse forebrain	Over-expression	Obesity, impaired glucose tolerance, some defects in motor functions	Wu et al. (2011)
10	BRASTO mice	Over-expression	Enhanced response to ghrelin hormone	Satoh et al. (2010)

Hypothalamic SIRT1 protein levels increase on feeding, and SIRT1 suppresses the expression of orexigenic AgRP (Morton et al., 2006; Sasaki et al., 2010). Conditional knock-out of SIRT1 in AgRP neurons (loxP-flanked exon 4 of *Sirt1* with Cre recombinase driven by the *Agrp* promoter) decreased their responses to the hunger inducing hormone ghrelin (Dietrich et al., 2010). Imai and colleagues’ BRASTO mice with transgenic over-expression of SIRT1 in the brain (mouse *Sirt1* driven by mouse prion (PrP) promoter) had conversely an enhanced response to ghrelin (Satoh et al., 2010). On the other hand, conditional knockout of SIRT1 in POMC neurons (loxP-flanked exon 4 of *Sirt1* with Cre recombinase driven by the *Pomc* promoter) was shown to reduce energy expenditure and increase susceptibility to diet-induced obesity, as well as signaling processes induced by the satiety hormone leptin (Ramadori et al., 2010). In a recent report in which SIRT1 was conditionally overexpressed in mouse POMC or AgRP neurons (using *Rosa26* locus knockin and induction by *Pomc*- or *Agrp-Cre*), the authors observed that over-expressed SIRT1 prevented age-associated weight gain in complementary ways (Sasaki et al., 2014). Over-expression of SIRT1 in POMC neurons stimulated energy expenditure, whereas overexpression in AgRP neurons suppressed food intake. Mice with brain-specific SIRT1 knock-out (BSKO: loxP-flanked exon 4 of *Sirt1* with Cre recombinase driven by the *nestin* promoter) have defective somatrophic (growth hormone and IGF-1) signaling (which could be due to *Sirt1’s* recently documented inhibition of CREB Monteserin-Garcia et al., 2013), as well as CR/DR response (Cohen et al., 2009).

SIRT1’s regulation of energy (glucose and lipid) metabolism in peripheral tissues are well known (Chalkiadaki and Guarente, 2012; Li, 2013), and SIRT1 has been associated with induction of hepatic gluconeogenesis (Rodgers et al., 2005), pancreatic insulin secretion (Bordone et al., 2006), enhanced muscle insulin sensitivity (Schenk et al., 2011) and the “browning” of white adipose tissues (Qiang et al., 2012). The SIRT1 activator resveratrol when acutely administered into the mediobasal hypothalamus (containing the arcuate nucleus) improves insulin sensitivity, and this is negated by SIRT1 inhibition or silencing (Knight et al., 2011). Conditional deletion of SIRT1 in mice in steroidogenic factor 1 (SF1)-expressing neurons (loxP-flanked exon 4 of *Sirt1* with Cre recombinase driven by the *Sf1* promoter of the ventromedial hypothalamic nucleus (VMH) resulted in heightened susceptibility to diet-induced obesity, and conversely mice overexpressing SIRT1 in these neurons are more resistant to diet-induced obesity and insulin resistance (Ramadori et al., 2011). It appears that SIRT1 activities in the hypothalamus are generally associated with a metabolically favorable phenotype that resembles that observed with CR/DR. On the other hand, it has also been reported that SIRT1 neuron specific knockout mice (with Cre recombinase controlled by of rat synapsin I promoter) had increased insulin sensitivity and increased insulin receptor signaling in peripheral tissues (Lu et al., 2013). Over-expression of SIRT1 in the mouse forebrain was also shown to result in obesity, impaired glucose tolerance and some defects in motor function (Wu et al., 2011). These deviations attest to the underlying complexities of the phenotypes resulting from pharmacological and genetic manipulations, which is dependent on the mice’s genetic background and the exact nature of manipulation.

**Figure 3:**
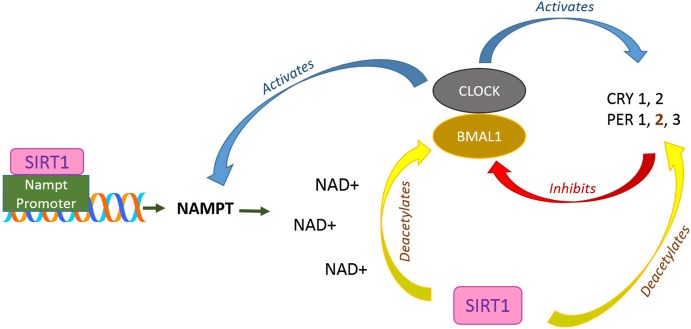
**SIRT1 roles in circadian rhythm regulation**. In the supraschiasmatic nucleus (SCN) at hypothalamus, CLOCK-BMAL1 activates the transcription of Cryptochrome (CRY) protein 1, 2 and Period (PER) 1, 2, and 3 which in turn, directly repress CLOCK-BMAL1 activity. In addition, CLOCK-BMAL1 also regulates circadian expression of NAMPT. Meanwhile, SIRT1 is recruited to the NAMPT promoter and contributing to the synthesis of its own coenzyme, NAD+, which is important for its deacetylation activity. SIRT1 also directly contributes to circadian rhythms through deacetylation of BMAL1 and PER2.

Another important aspect of SIRT1 activity which is tightly connected to metabolic regulation that has recently come to light is its role in the regulation of circadian rhythm (Maury et al., 2010; Rey and Reddy, 2013). Central control of the mammalian circadian rhythm resides in the hypothalamic suprachiasmatic nucleus (SCN), and is regulated by a feedback loop of transcription factor interactions. Brain and muscle Arnt-like protein 1 (BMAL1) and CLOCK activate the expression of PERIOD 1, 2 and 3 (PER1, 2, 3) and Cryptochrome 1 and 2 (Cry1, 2) genes, with the latter complexing at high levels with the former, repressing their own transcription. The central circadian timer protein CLOCK is a histone acetyltransferase, and SIRT1 deacetylase activity appears to antagonize it in a circadian manner (Nakahata et al., 2009). In peripheral tissues, SIRT1 binds CLOCK-BMAL1 and is thus recruited to the CLOCK-BMAL1 chromatin complex at circadian gene promoters (Nakahata et al., 2008). It could deacetylate BMAL1 to affect its transcriptional activity (Nakahata et al., 2008), and it could also deacetylate and promote the degradation of PER2 (Asher et al., 2008). Clock-Bmal1 regulates the circadian expression of nicotinamide phosphoribosyltransferase (NAMPT), the rate-limiting enzyme in the NAD^+^ salvage pathway (Nakahata et al., 2009; Ramsey et al., 2009), which is in turn important for SIRT1 activity. While CLOCK promotes NAMPT expression, inhibition of SIRT1, or of NAMPT by FK866, promotes an early onset of circadian peak of circadian genes like *Per2* and *Dbp* (Nakahata et al., 2009). Thus, there appears to be a feedback loop of CLOCK-BMAL1 transcriptional regulation of clock genes involving NAMPT/NAD^+^ and SIRT1 (Figure 3).

An important notion that emerges and extended from the above findings is that an essential aspect of SIRT1’s role in aging and aging-associated metabolic diseases lies in its activity at the hypothalamus. As mentioned above, BRASTO mice generated by Imai’s group had metabolic and aging parameters that are superior to age-matched controls, and demonstrated a delayed-aging phenotype and an increased lifespan (Satoh et al., 2013). The phenotype of BRASTO mice was attributed to an enhanced neural activity (as access by *c-Fos* expression) and the expression of orexin type 2 receptor gene *Ox2r* at the dorsomedial and lateral hypothalamic nuclei, and not the arcuate and ventromedial hypothalamic nuclei in aged BRASTO mice. *Ox2r* promoter activity was enhanced by SIRT1 expression through deacetylation of the transcription factor Nk2 homeobox1 (NKX2-1), and Sirt1-NKX2-1-Ox2r was proposed by the authors to form a functional axis responsible for the delayed-aging and increased lifespan phenotype. Interestingly, the phenotypes could not be directly or causally related to SIRT1 expression in the arcuate nuclei, which influences food intake and energy metabolism. Another BRASTO transgenic line with SIRT1 expression that is much higher throughout the brain and homogeneously higher throughout the hypothalamus does not display delayed-aging or increased lifespan (Satoh et al., 2013).

SIRT1’s regulation of the central control of circadian rhythm is affected by aging. At the hypothalamic SCN, neuronal SIRT1 and PGC-1α bind cooperatively and in close proximity at the Bmal1 promoter to regulate Bmal1 expression (Chang and Guarente, 2013). In aged mice, decreased SIRT1 levels in the SCN and reduced Bmal1 and Per2 perturbs the circadian rhythm and impairs the ability to adapt to environmental light/dark changes, a phenotype that is also exhibited by conditional SIRT1 knock-out in young mice. Overexpression of SIRT1 in the brain counters the aging-associated circadian rhythm defects. As impair circadian rhythm contributes critically to metabolic disorders, this result suggests that SIRT1’s activity at the SCN is important for delaying the onset of metabolic dysfunction that result from impaired central control of circadian rhythm.

## SIRT1’s role in CNS neuroprotection and neurodegeneration

SIRT1 has been associated with a neuroprotective function in a myriad of neuronal injury and neurodegeneration paradigms (Gan and Mucke, 2008; Pallàs et al., 2008; Tang, 2009; Donmez and Outeiro, 2013; Herskovits and Guarente, 2014). A large number of studies using genetic and pharmacological manipulation of SIRT1 activity in invertebrates and mice models of neuronal injuries and disorders have been reported. Interestingly, both activation and inhibition of SIRT1 have been shown to be beneficial. It should also be noted that SIRT1 activities are not always pro-survival (Li et al., 2008; Ng and Tang, 2013; Sansone et al., 2013). SIRT1 activation by resveratrol (Raval et al., 2006) or transgenic overexpression of the protein (Hernández-Jiménez et al., 2013) was shown to protect against brain ischemic damages, as well as neuronal death in traumatic brain injury (Zhao et al., 2012). On the other hand, the SIRT1 inhibitor nicotinamide could also protect neurons against excitotoxicity and cerebral ischemia (Liu et al., 2009). An early study showed that resveratrol protected both *C. elegans* and mouse neurons against the cytotoxicity of mutant huntingtin (Parker et al., 2005). Later genetic manipulations in mice appeared to confirm SIRT1’s beneficial effect in Huntington’s disease (HD; Jeong et al., 2011; Jiang et al., 2011), but the SIRT1 inhibitor selisistat was recently shown to alleviate HD pathology in *Drosophila* and mouse models (Smith et al., 2014). The beneficial effect of SIRT1 elevation or activation for models of amyotrophic lateral sclerosis is more consistent (Kim et al., 2007; Song et al., 2014). SIRT1 activity is also generally shown to protect against toxic α-synuclein aggregates in worm and mice models (van Ham et al., 2008; Donmez et al., 2012), and protected dopaminergic neurons in the MPTP mouse model of Parkinson’s disease (Mudò et al., 2012).

As far as aging and aging-associated disorder is concerned, however, the main neurodegenerative disease of interest would be Alzheimer’s disease (AD; Ballard et al., 2011; Bonda et al., 2011) and related dementias, which are discussed below.

Multiple evidences indicate that SIRT1 elevation or activation is beneficial in AD and dementia (Braidy et al., 2012; Lalla and Donmez, 2013). SIRT1’s attenuation of NF-қB signaling in microglia was shown to be protective against neuronal death induced by Aβ peptides (Chen et al., 2005). This role of SIRT1 in AD is apparently connected to CR/DR, which reduces Aβ generation and amyloid plaque deposition in the brain of Tg2576 transgenic AD mice (Wang et al., 2005) and primate (Qin et al., 2006a). SIRT1 activation by resveratrol was shown to protect against toxicity induced by the cyclin-dependent kinase 5 (Cdk5) activator p25, and mutant Cu/Zn superoxide dismutase 1 (SOD1) in culture neurons and transgenic mice (Kim et al., 2007). Furthermore, direct injection of SIRT1-expressing lentivirus into the hippocampal CA1 region of p25 transgenic mice protected against aging associated neurodegeneration (Kim et al., 2007). In another AD mice model (bearing *App(swe)* and *Psen1 dE9* transgenes), transgenic over-expression of SIRT1 in the brain markedly reduced amyloid plaque formation, gliosis and decline in learning and memory capabilities, while brain SIRT1 specific knock-out had greatly attenuated lifespan likely due to juvenile onset of an AD-like disease (Donmez et al., 2010). However, contrasting the above, the SIRT1 inhibitor nicotinamide was also shown to attenuate/delay cognitive effects of 3xTg-AD mice (triple transgenic for mutant *Psen1*, *App(swe)* and *Tau*) via SIRT1 inhibition and reduction of Tau phosphorylation (Green et al., 2008).

The mechanisms underlying the protective effect of SIRT1 and its activity in AD models are complex and multifaceted. SIRT1’s reduction of Aβ generation appears unique to AD pathology, and has thus been the focused of several studies that yielded interesting and useful insights. Aβ production requires the sequential action of β-secretase BACE1 and the γ-secretase complex on the amyloid precursor protein (APP). If APP is first cleaved by an α-secretase, a member of the A Disintegrin and metalloproteinase (ADAM) family, its BACE1 recognition site is loss. Aβ production is effectively prevented (Bandyopadhyay et al., 2007), and the product of α-secretase cleavage, the soluble N-terminal fragment of APP (sAPPα), is neuroprotective (Corrigan et al., 2012). SIRT1 over-expression in primary neuron cultures from Tg2576 mouse reduced Aβ secretion resulted in elevated levels of sAPPα and decreased Rho-activated kinase 1 (ROCK1) expression. This mimicked the phenotype elicited by caloric restriction (Qin et al., 2006b). Incidentally, blood cholesterol-lowering inhibitors of 3-hydroxy-3-methyl-glutaryl (HMG)-CoA reductase (statins) have also been shown to improve cognitive function and amyloid phenotype of AD models in multiple studies (Tong et al., 2009; Kurata et al., 2011). Statins also increased sAPPα production (Pedrini et al., 2005), which likely occurs via inhibition of ROCK signaling, as statins also inhibit isoprenoid biosynthesis that would affect the activity of Rho family GTPases upstream of ROCK (Ma et al., 2009). A constitutively-active ROCK1, which inhibited statin-stimulated sAPPα shedding, could in fact attenuate the SIRT1-mediated response (Qin et al., 2006b). Recent studies have indicated that the influence of Rho/ROCK signaling on amyloidogenesis is complex, involving the phosphorylation of APP and modulation of BACE1 activity (Herskovits and Guarente, 2014).

**Figure 4:**
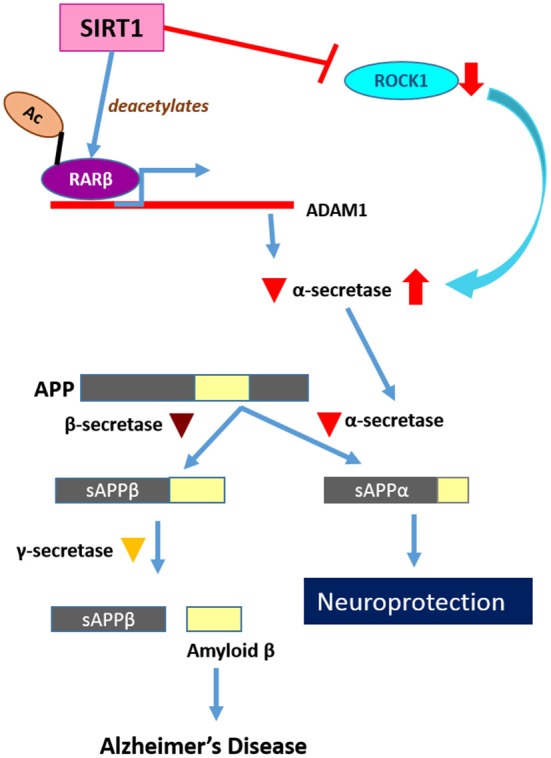
**SIRT1’s roles in neuroprotection and neurodegeneration**. SIRT1 has a neuroprotective function through suppression of amyloid β production. SIRT1 activates RARβ as a transcription factor of ADAM10, which encodes α-secretase. The APP cleaved by α-secretase is no longer able to generate Amyloid β peptides and this non-amyloidogenic cleavage is therefore neuroprotective.

A more recent report has shown that SIRT1 could directly activate the transcription of the α-secretase ADAM10, likely via deacetylation of retinoic acid receptor β (RARβ), which is known to activate ADAM10 transcription (Donmez et al., 2010; Figure 4). SIRT1 over-expression in mouse neural N2a cells expressing mutant APPswe have significantly elevated ADAM10 and sAPPα levels, and cilostazol (an inhibitor of type III phosphodiesterase) appears to suppress Aβ production in a SIRT1-RAR-ADAM10-dependent manner (Lee et al., 2014). SIRT1’s protective effect of AD-susceptible neurons may be preceded by enhanced α-secretase-mediated non-amyloidogenic APP processing, and a decline in SIRT1 levels in the aged brain would therefore predispose its neurons to amyloidogenic APP processing and AD. Not much yet is known about how cholesterol and isoprenoid metabolism affects development and progression of AD (Sun et al., 2014; Wood et al., 2014). However, other connections between SIRT1, cholesterol metabolism and AD exist. The apolipoprotein E ε4 allele (ApoE4), a major risk factor for AD (Hauser and Ryan, 2013), binds to APP and significantly reduces sAPPα secretion, sAPPα/Aβ, and sAPPα/sAPPβ ratios. In cell culture and AD postmortem tissue, ApoE4 expression is shown to be associated with a decreased level of SIRT1 and increased level of SIRT2 (which is associated with neurodegeneration Liu et al., 2012; Outeiro et al., 2007), resulting in a decrease in SIRT1/SIRT2 ratio (Theendakara et al., 2013). The decreased SIRT1/SIRT2 ratio is perhaps indicative of a neuroprotective state that has been reversed by ApoE4 expression.

## Epilogue

In the discussions above, we highlighted known and recently revealed roles and activities of SIRT1 in the brain. Many previous reports have attested to an improved metabolic status and health at late stages of life with SIRT1 elevation and activation (reviewed in Cantó and Auwerx, 2009a; Chaudhary and Pfluger, 2009; Chang and Guarente, 2014), but evidence for actual lifespan extension by SIRT1 is only recently reported (Satoh et al., 2013; Mercken et al., 2014; Mitchell et al., 2014). We expounded on the notion that SIRT1’s role at the late stages of life and lifespan itself are linked with, and causally related to, its activities in the brain. In particular, two key aspects of late stages of life that are dependent on SIRT1 are normally loss, namely cognitive function and metabolic regulation. The three strongest lines of evidence in this regard are (1) SIRT1 is important for certain aspects of neural connectivity and synaptic plasticity, (2) Loss or decline in CNS SIRT1 activity occurs during aging, and this deficit is directly associated with defined neurophysiological and neuropathological mechanisms of cognitive decline and metabolic dysfunction; and (3) SIRT1 overexpression in certain neurons in the brain increased lifespan.

A more comprehensive understanding of SIRT1’s role in CNS neurons, particularly those of the hypothalamus and hippocampus, would hopefully aid future therapeutic interventions. However, much remains to be learned. Mouse experiments already calls for caution in analysis and interpretation. A constitutive and global elevation of brain SIRT1 (by whatever means) is unlikely to be helpful in an all-encompassing manner in reversing cognitive and metabolic dysfunctions. Notably, overexpression of brain SIRT1 in mice has been reported to result in metabolic (Wu et al., 2011) and cognitive (Kakefuda et al., 2009) defects. Our understanding of SIRT1’s regulation of circadian rhythm is at an early stage, and the roles of other nuclear sirtuins in this regard, such as SIRT6, are being revealed (Masri and Sassone-Corsi, 2014). SIRT6 also interacts with CLOCK-BMAL1, but probably not in the same complex as SIRT1. Unlike SIRT1, it operates directly at the transcription level by recruiting the clock machinery to chromatin. An important role for SIRT6 is circadian chromatin recruitment of SREBP-1, thus impinging on the circadian regulation of a host of genes in fatty acid and cholesterol metabolism (Masri et al., 2014). A thorough understanding of how SIRT1 and SIRT6 differentially or synergistically regulate the circadian mechanism would be of fundamental importance (Masri and Sassone-Corsi, 2014).

An other important aspect of note is that beyond its role in regulating gene expression via deacetylation of transcription factors, Sirt1 is also a regulator of epigenetic changes. Sirt1 is a histone deacetylase (Hayakawa et al., 2015), and could alter DNA methylation through its activity on factors such as MeCP2 (Zocchi and Sassone-Corsi, 2012). A recent report in mice showed that microglial SIRT1 deficiency influences both aging-associated and tau-mediated memory deficits via the pro-inflammatory cytokine IL-1β (Cho et al., 2015). Activation of IL-1β transcription by SIRT1 deficiency appears to result from IL-1β proximal promoter hypomethylation, a state that is strongly associated with elevated IL-1β expression and aging. A more defined understanding of the epigenetic impact of SIRT1, either direct or indirect, on organismal aging via the neurons would be highly desirable. On the other hand, molecules linking SIRT1 to learning and memory are not yet fully explored. The histone H2A variant, H2A.Z, a known mediator of thermosensory response, was recently shown to play a role in memory consolidation (Zovkic et al., 2014). Interestingly, at least in some tissues, SIRT1 level and activity negatively affect H2A.Z expression (Baptista et al., 2013). It is therefore plausible that SIRT1’s role in learning and memory is connected to its effect on H2A.Z expression, a notion that requires further confirmation.

The mitochondrial SIRT3, SIRT4 and SIRT5 could also be key regulators of metabolism (Hirschey et al., 2010; Laurent et al., 2013a,b; Rardin et al., 2013; Weir et al., 2013), and the interplay or crosstalk between nuclear SIRT1 and mitochondrial SIRT3 in metabolic regulation and disorder is not yet understood in detail. Sirtuin-mediated lifespan extension has now been observed for SIRT1, SIRT6 (Kanfi et al., 2012), and under specific circumstances, for SIRT2 (North et al., 2014). An understanding of how mammalian sirtuins interact in complex traits like lifespan would require much more work.

SIRT1’s neuroprotective function has also been extended by recent findings, and it appears to play a role in the maintenance of genomic stability in postmitotic neurons (Dobbin et al., 2013). SIRT1 deacetylates and activates HDAC1 in the repair of double stranded DNA breaks through the nonhomologous end-joining pathway. In as far as learning and memory is concern, SIRT1 and HDACs may have, albeit indirect, opposite functions. While SIRT1 activation preserves memory and cognition (Gao et al., 2010; Michán et al., 2010), HDAC inhibition enhances cognition (Gräff and Tsai, 2013). HDAC inhibitors are known to have varying effects on the levels of sirtuins, and trichostatin A (TSA) and butyrate downregulate Sirt1 (Kyrylenko et al., 2003). How sirtuins interact functionally with HDACs in their myriad neuronal functions would also be an interesting and important pursuit in the near future.

## Conflict of interest statement

The authors declare that the research was conducted in the absence of any commercial or financial relationships that could be construed as a potential conflict of interest.
